# Data Center Four-Channel Multimode Interference Multiplexer Using Silicon Nitride Technology

**DOI:** 10.3390/nano14060486

**Published:** 2024-03-08

**Authors:** Ophir Isakov, Aviv Frishman, Dror Malka

**Affiliations:** Faculty of Engineering Holon, Institute of Technology (HIT), Holon 5810201, Israel

**Keywords:** multimode interference, silicon nitride, multiplexer, WDM

## Abstract

The operation of a four-channel multiplexer, utilizing multimode interference (MMI) wavelength division multiplexing (WDM) technology, can be designed through the cascading of MMI couplers or by employing angled MMI couplers. However, conventional designs often occupy a larger footprint, spanning a few millimeters, thereby escalating the energy power requirements for the photonic chip. In response to this challenge, we propose an innovative design for a four-channel silicon nitride (Si_3_N_4_) MMI coupler with a compact footprint. This design utilizes only a single MMI coupler unit, operating within the O-band spectrum. The resulting multiplexer device can efficiently transmit four channels with a wavelength spacing of 20 nm, covering the O-band spectrum from 1270 to 1330 nm, after a short light propagation of 22.8 µm. Notably, the multiplexer achieves a power efficiency of 70% from the total input energy derived from the four O-band signals. Power losses range from 1.24 to 1.67 dB, and the MMI coupler length and width exhibit a favorable tolerance range. Leveraging Si_3_N_4_ material and waveguide inputs and output tapers minimizes light reflection from the MMI coupler at the input channels. Consequently, this Si_3_N_4_-based MMI multiplexer proves suitable for deployment in O-band transceiver data centers employing WDM methodology. Its implementation offers the potential for higher data bitrates while maintaining an exemplary energy consumption profile for the chip footprint.

## 1. Introduction

The demand for bandwidth and data throughput in optical data centers is increasing rapidly, and this requires the development of compact and high-performing multiplexing devices [1,2]. This research paper proposes a new four-channel multiplexer that is specifically designed for the O-band spectrum. The study introduces a unique single MMI coupler unit, which is different from traditional wavelength division multiplexer (WDM) designs that rely on multiple MMI couplers [3,4,5,6,7,8]. The proposed approach is designed to mitigate space constraints, a crucial consideration in densely packed optical data centers, while maintaining high operational efficiency [9]. The innovative design addresses space constraints and simplifies the manufacturing process, potentially leading to more cost-effective and scalable optical network solutions. The O-band spectrum notably offers significantly improved signal integrity over extended distances, a critical feature essential for the evolving demands of modern data center networks [10]. The multiplexer’s design is based on the principle of multimode interference (MMI), which allows for the uniform distribution of light from multiple input ports toward the output port via the self-imaging effect [11,12,13]. The design also takes advantage of silicon nitride (Si_3_N_4_), a material with low propagation loss, high thermal stability [14], and compatibility with standard complementary metal-oxide-semiconductor (CMOS) manufacturing processes [15,16], making it an ideal material for integrated photonic applications.

MMI devices are crucial in various photonic applications, including in the multiplexing and demultiplexing of optical signals [17]. The MMI phenomenon is rooted in the self-imaging principle. This principle involves multiple modes of light propagating within a multimode waveguide and undergoing constructive interference at periodic intervals, thereby reproducing the input light pattern at the output [18]. In the context of wavelength division multiplexing (WDM) systems, this capability is indispensable. WDM systems utilize diverse wavelengths to carry different data streams [19], and MMI multiplexers are adept at combining or segregating these wavelengths [20]. This functionality significantly increases the data capacity of single optical fibers, making them a cornerstone of modern high-bandwidth data centers.

Si_3_N_4_ is selected for the waveguide core due to its favorable characteristics in the O-band spectrum, including low propagation loss and high refractive index contrast with silicon dioxide (SiO_2_) cladding [21]. Si_3_N_4_ has a low material absorption coefficient in the near-infrared to the mid-infrared regions, which translates to low propagation losses [22]. Empirically, propagation losses as low as 0.1 dB/cm have been reported in the literature for Si_3_N_4_ waveguides [23], which are significantly lower than those of many other waveguide materials such as Si [24]. These properties are instrumental in achieving efficient light confinement and low crosstalk, essential for the multiplexer’s performance. The high refractive index contrast with the SiO_2_ results in strong optical confinement [25], enabling tight bends and compact device geometries, which are essential for densely packed photonic circuits.

Moreover, the compatibility of Si_3_N_4_ with CMOS technology simplifies the integration process with existing semiconductor electronics [26,27], meaning it can be seamlessly incorporated into existing semiconductor manufacturing processes [28]. This compatibility streamlines the fabrication of optical circuits, allowing for the co-integration of electronic and photonic components on a single chip [29], which is a game-changer for developing multifunctional, high-density optical chips. The transition to Si_3_N_4_ from conventional materials like silicon on insulators represents a paradigm shift, enabling higher power handling [30], reduced nonlinear effects [31], and improved thermal stability [32], thereby enhancing the overall reliability and performance of photonic integrated circuits [33].

One of the most critical aspects of waveguide design is the mitigation of back reflection [34], which can lead to signal degradation and instability in laser sources [35]. Back reflections arise from mismatches within the waveguide, leading to a portion of the light being reflected back towards the source [36]. Si_3_N_4_ offers an intrinsic advantage in this regard [37], demonstrating inherently low back reflection [38,39], which is further optimized through the careful design of waveguide tapers [40]. This characteristic is crucial in maintaining the integrity of the signal and ensuring the reliable operation of photonic devices, particularly in systems where wavelength stability and signal clarity are paramount.

This paper introduces a novel design for a 4 × 1 MMI Multiplexer employing Si_3_N_4_ waveguides, optimized to operate within the O-Band spectrum—a region renowned for its low fiber dispersion and attenuation. The unique characteristics of Si_3_N_4_ are utilized to create a compact footprint, essential for integrating photonic components in applications where space is limited, such as data centers. The proposed multiplexer is meticulously optimized to merge four discrete optical signals ranging from 1270 to 1330 nm with 20 nm spacing efficiently into a single output, thus serving as a cornerstone for the next generation of high-speed optical interconnects.

## 2. The 4 × 1 Power Multiplexer Structure and the Theoretical Aspect

The O-Band 4 × 1 power multiplexer is a pivotal component in advanced optical data center systems, enabling the integration of four distinct optical signals into a single output waveguide. It plays a vital role in applications such as WDM, where multiple signals at different wavelengths need to be combined for transmission over a single fiber. The multiplexer employs Si_3_N_4_ for the waveguide core due to its optimal properties in the O-band spectral region, including low propagation loss and a high refractive index contrast with SiO_2_ cladding. These characteristics are crucial for achieving efficient light confinement and low crosstalk in a compact footprint.

The multimode interference (MMI) coupler is the main component of the multiplexer. It is designed to exploit the MMI phenomenon, where several guided modes can coexist and interfere within the waveguide, allowing for the distribution of optical power from multiple input ports to a single output. Each input waveguide carries a specific wavelength, and the multiplexer combines these channels into a composite signal.

**Figure 1:**
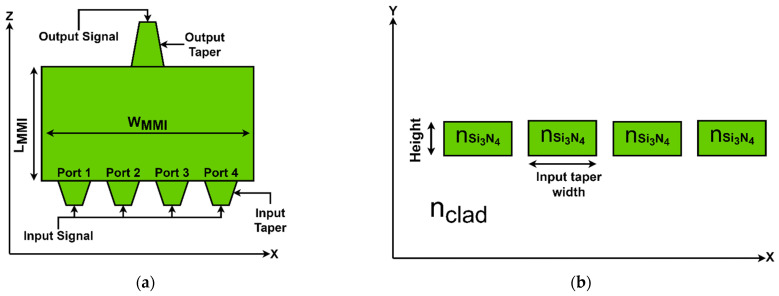
Optical 4 × 1 MMI coupler multiplexer: (**a**) x–z cross-section; (**b**) x–y cross-section.

Figure 1a displays the cross-section of the 4 × 1 O-Band multiplexer in the x–z plane, showing the detailed configuration of its components. The white shaded areas represent the SiO_2_ cladding material, with a refractive index of 1.447. The green-colored area represents the Si_3_N_4_ waveguide core, characterized by a higher refractive index, as delineated in Table 1, which lists the refractive indices corresponding to the operating wavelengths. This design integrates the input and output tapers with a central MMI coupler, with each component being integral to the device’s functionality. The input tapers are designed with precision, extending 4.4 µm in length and transitioning in width from 0.8 µm to 1 µm to facilitate optimal mode coupling into the MMI region. The output taper, responsible for channeling the combined signal, measures 7 µm in length, and it has a variable width, starting at 1.6 µm at its broadest point and tapering down to 0.8 µm. Each port is dedicated to a distinct wavelength, with port 1 aligned to 1310 nm, port 2 to 1290 nm, port 3 to 1270 nm, and port 4 to 1330 nm, covering a broad range within the O-Band spectrum. Figure 1b exhibits the Si_3_N_4_ strip waveguide structures in an x–y cross-section at the z = 0 µm point.

The multiplexer operates by using self-imaging and total internal reflection (TIR) principles. The self-imaging effect within the MMI region is of utmost importance for its operation, merging multiple input signals into a coherent output. Simultaneously, the TIR effect ensures effective light confinement within the waveguide, which is crucial for the efficient transmission of optical signals, reducing energy losses and maintaining the clarity and strength of the signals while they are merged and transmitted through the multiplexer.

The beat length, denoted as *L_π_*, is a key parameter that determines the operation of the MMI coupler within the multiplexer. It represents the distance over which the first two modes propagating through the MMI region exhibit a phase difference of π radians. Approximating *L_π_* and analyzing waveguide parameters helps optimize the multiplexer’s design for efficient power distribution and minimal signal degradation. An approximate expression for *L_π_* is given by

(1) $$L_{\pi }\approx \;\frac{4n_{eff}W_{e}^{2}}{3\lambda }$$

Here, *n_eff_* is the effective refractive index, critical for mode propagation and design optimization. The operational wavelengths, *λ*, are 1.27 µm, 1.29 µm, 1.31 µm, and 1.33 µm. The effective width (*W_e_*) of a waveguide is an important parameter that influences the mode confinement and propagation characteristics within the waveguide. The expression for *W_e_* is

(2) $$W_{e}=W_{MMI}+\frac{\lambda }{\pi }{\left(\frac{n_{clad}}{n_{eff}}\right)}^{2\sigma }\frac{1}{\sqrt{{n_{eff}}^{2}-{n_{clad}}^{2}}}$$

Here, *W_MMI_*, represents the width of the MMI region. It determines the extent of mode interaction and interference effects within the waveguide structure. This equation accounts for the phase difference between adjacent modes and influences the periodicity and self-imaging effect within the waveguide. In the case of TE polarization, σ = 0, and σ = 1 for TM. The overall length of the MMI region, *L_MMI_*, is calculated using

(3) $$L_{MMI}=\frac{3pL_{\pi }}{4N}$$

This equation integrates the beat length *L_π_*, the number of input sections *N*, and a numerical factor *p*, typically set to 1, which accounts for the MMI structure’s specific configuration and design. The length of the MMI region is proportional to these factors and is vital for optimizing the performance of MMI-based waveguide devices.

The innovative design of our single MMI coupler is predicated on a specific length condition that allows for its proper functioning across multiple wavelengths. The *L_MMI_* must satisfy the following relationship to ensure that the input signals at different wavelengths interfere constructively within the MMI and are efficiently combined into the output port:

(4) $$L_{MMI}=p_{1}L_{\pi }^{\lambda 1}=\left(p_{2}+q_{2}\right)L_{\pi }^{\lambda 2}=\left(p_{3}+q_{3}\right)L_{\pi }^{\lambda 3}=\left(p_{4}+q_{4}\right)L_{\pi }^{\lambda 4}$$

where *p* is a positive integer, *q* is an odd integer and $L_{\pi }^{\lambda }$ is the beat length at specific wavelength *λ*. Finally, the evaluation of the multiplexer’s performance includes the analysis of insertion losses at the output ports. These losses are critical in determining how much signal power is lost during the merging process. The insertion loss, *IL*, can be quantified using

(5) $$IL=-10\log_{10}\frac{P_{out}}{P_{in}}$$

where *P_in_* is the input power and *P_out_* is the output power. This measure is instrumental in assessing the efficiency of the power-merging process and the overall performance of the device. Lower insertion losses signify better power transfer.

## 3. Simulation Results

Utilizing Rsoft photonic CAD software (The RSoft CAD Environment™), simulations were conducted on the MMI multiplexer coupler and the Si_3_N_4_ strip waveguide structure using the beam propagation method (BPM) and finite-difference time-domain (FDTD) tools. Subsequently, Matlab scripts were employed to analyze the results and identify optimal values.

**Figure 2:**
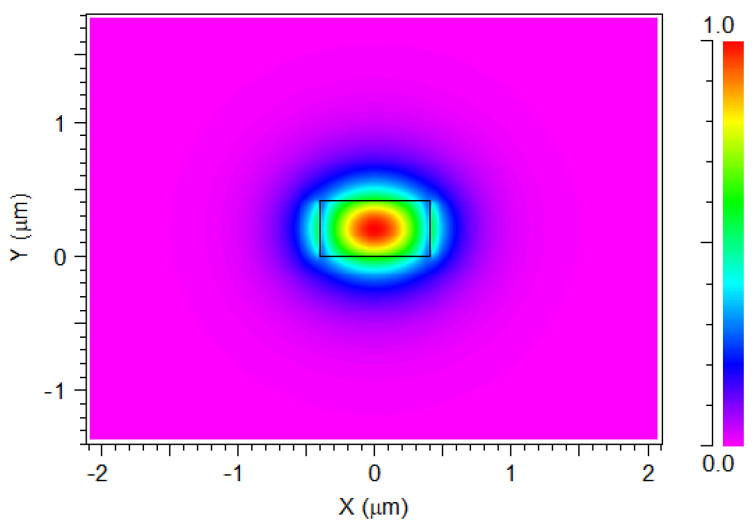
The TE fundamental mode profile at 1310 nm wavelength.

Figure 2 shows the TE fundamental mode profile within the Si_3_N_4_ strip waveguide at the XY plane for an operational wavelength of 1310 nm. In this figure, the red color highlights the area of high light confinement inside the Si_3_N_4_ strip waveguide area of 410 × 800 nm. The quasi-TE fundamental mode exhibits a distinctive field profile, with different regions experiencing varying levels of electric field intensity. This mode profile is crucial in analyzing and optimizing the performance of the waveguide for specific applications. By examining the color-coded visualization, it is possible to observe regions with strong power confinement, indicated by higher intensity regions, typically represented by vibrant colors such as red. These regions demonstrate effective localization and strong power propagation within the waveguide. The exact same mode profile behavior is observed for the operational wavelengths at 1270, 1290, and 1330 nm. Therefore, to avoid redundancy, only one solution for 1310 nm is shown.

**Figure 3:**
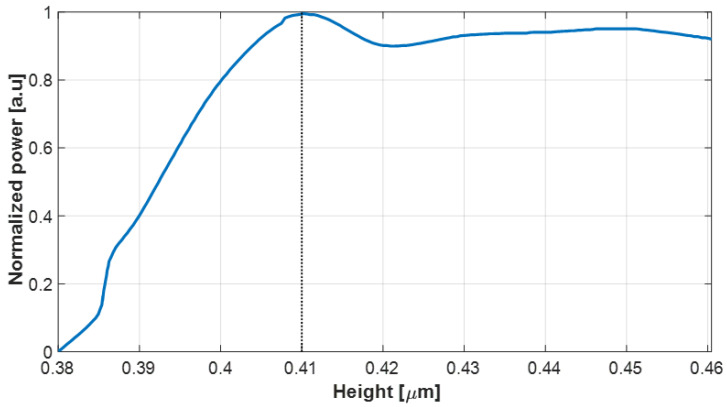
Normalized power as a function of the Si_3_N_4_ strip waveguide height.

Figure 3 shows the optimal height of the Si_3_N_4_ strip waveguide, which is 410 nm, in the design of the MMI multiplexer. It is important to note that heights exceeding 0.41 µm do not lead to a major decrease in power, and the normalized power was maintained above 90% for any increase in height up to 0.46 µm. However, increasing the thickness of the Si_3_N_4_ layer introduces several challenges and concerns. One such challenge is the potential increase in propagation loss. The Si_3_N_4_ thickness layer can result in higher optical losses, reducing the overall efficiency of the waveguide device. This factor needs to be carefully considered to maintain optimal signal transmission. Additionally, it is evident that a favorable tolerance range of ±10 nm is achieved around the optimal value, accounting for 90% of the total normalized power. This proves beneficial in addressing minor deviations in the Si_3_N_4_ thickness layer that may occur during the fabrication process.

**Figure 4:**
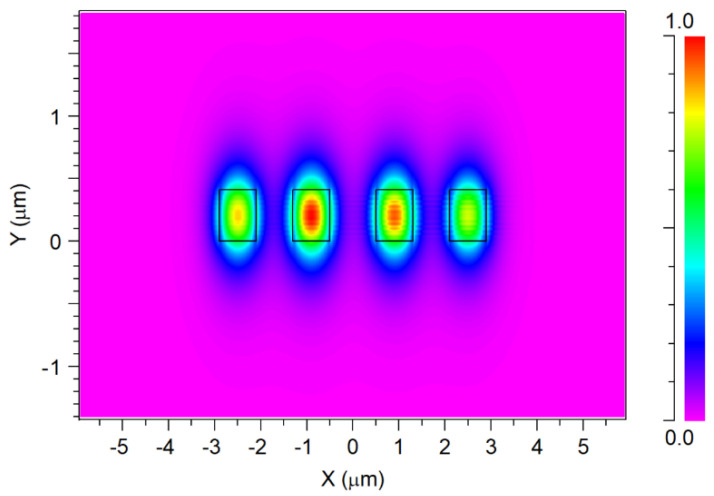
Quasi-TE fundamental mode profile for each operated wavelength at the input taper waveguides.

The selection of specific geometrical values of the input taper and the gap values between the four ports were carefully made to promote a robust confinement of the electric fields of the four signals, avoiding any coupling light between the four input signals within the input taper waveguide region. The successful achievement of this desired light confinement is clearly shown in Figure 4, where each mode electric field of the optical signal within the input taper waveguides region can be seen without any light coupling between the four signals and the optimal gap values between are 1.2 µm (between port 2 and port 3) and 0.7 µm (between ports 1/3 and ports 2/4).

**Figure 5:**
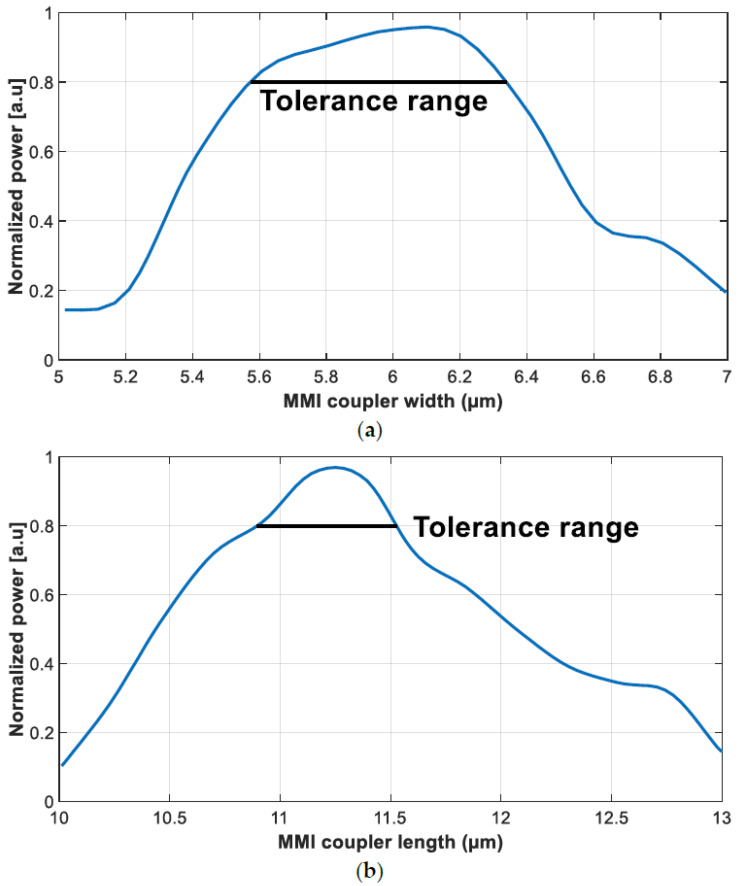
Normalized power as function of the geometrical MMI coupler parameters: (**a**) *W_MMI_*; (**b**) MMI coupler length (*L_MMI_*).

The normalized power in the output of the MMI coupler can be influenced by its geometrical parameters, specifically the MMI coupler width (*W_MMI_*) and length (*L_MMI_*). Figure 5a,b show the MMI coupler optimizations between the normalized power and these respective parameters. Figure 5a,b illustrate the optimized values for the *W_MMI_* and *L_MMI_* structures, respectively. The optimal width for the *W_MMI_* is determined to be 6 µm, while the *L_MMI_* has an optimized length of 11.2 µm. These values have been meticulously chosen to guarantee the optimal performance and efficient operation of the proposed multiplexer, considering potential minor fabrication errors that may occur in a high-quality fabrication facility, typically within a range of ±20 nm from the optimal values. In Figure 5a, the normalized power is plotted against the width (*W_MMI_*) of the MMI coupler. It illustrates how varying the width within a certain range impacts the power transmission efficiency. This provides insights into the optimal width required to achieve maximum power transfer and minimize power losses. To account for manufacturing tolerances and variations, tolerance ranges have been defined. For the *W_MMI_*, the tolerance range spans 5.55 µm to 6.35 µm with 80% of the total normalized power, allowing a large variation in width of +350 nm and −450 nm from the optimal value while maintaining the desired performance. Figure 5b shows the normalized power as a function of the length (*L_MMI_*) of the MMI coupler. It demonstrates the influence of the length on the power distribution and propagation characteristics within the coupler. The tolerance range for the *L_MMI_* is between 10.9 µm and 11.5 µm with 80% of the total normalized power, as can be seen in Figure 5b. These findings provide valuable insights into the precise design specifications necessary to fabricate the MMI coupler, which in our case has large stability regarding error fabrication because of the larger tolerance range of ±300 nm.

**Figure 6:**
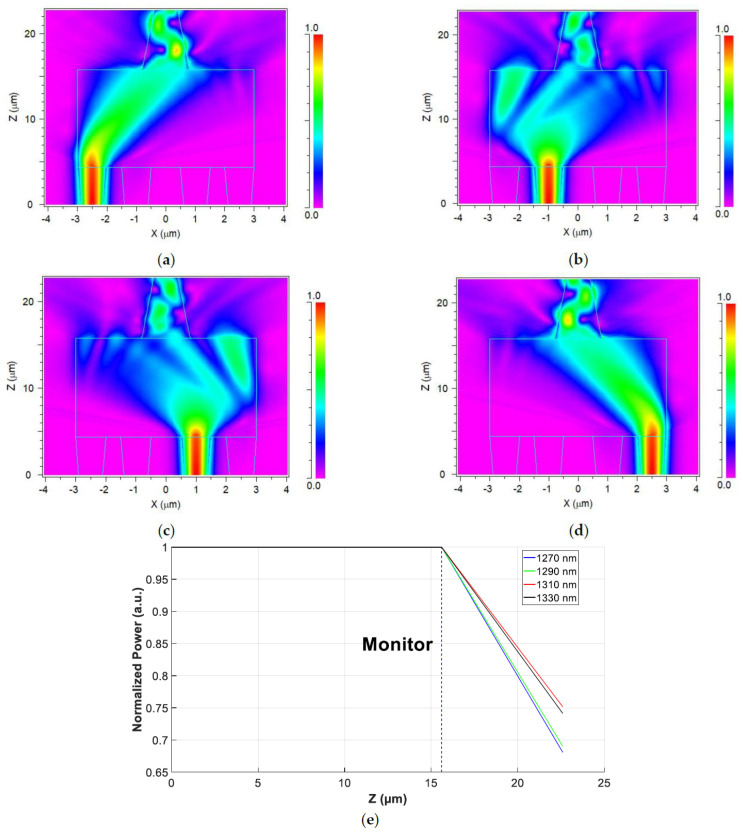
Intensity light profile for the operated wavelengths and insertion losses: (**a**) 1310 nm (port 1); (**b**) 1290 nm (port 2); (**c**) 1270 (port 3); (**d**) 1330 nm (port 4). (**e**) Propagation losses over the device length.

Figure 6a–d show the intensity profile for each operated wavelength in the proposed MMI multiplexer. Figure 6a illustrates the 1310 nm wavelength optical path from input port 1 to the output taper, with a 1.24 dB propagation power loss. Figure 6b shows the 1290 nm wavelength optical path from input port 2 to the output taper, with a 1.61 dB power propagation loss. Figure 6c shows the 1270 nm wavelength optical path from input port 3 to the output taper, featuring a 1.67 dB power propagation loss. Figure 6d shows the 1330 nm wavelength optical path from input port 4 to the output taper, with a 1.3 dB power propagation loss. Figure 6e shows the propagation loss for each operated wavelength. The monitor was placed at the output port.

**Figure 7:**
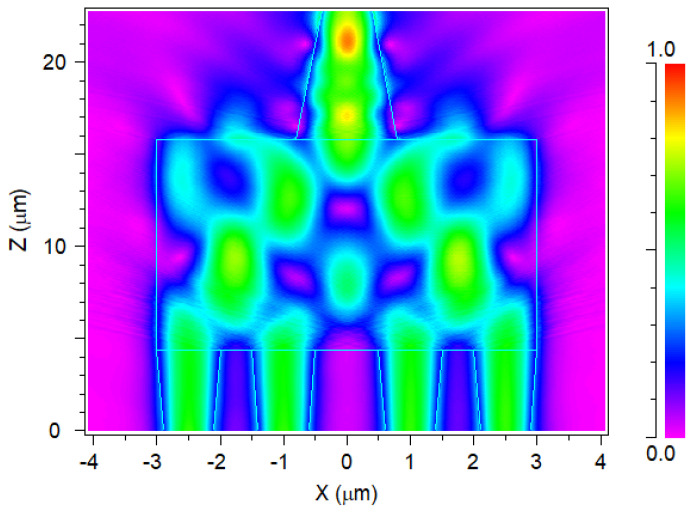
Intensity light profile for the 4 × 1 MMI multiplexer at the x–z plane.

Figure 7 demonstrates the operation of the 4 × 1 MMI multiplexer and its intensity profile for the four optical signals (1270 nm, 1290 nm, 1310 nm, 1330 nm), where each signal has a 0.3 normalized power level. The overall losses of the O-band 4 × 1 MMI multiplexer are observed to be 1.487 dB, indicating that approximately 30% of the total input power is lost due to the phase change that happens due to the self-imaging, resulting in light scattering within the MMI coupler, as shown in Figure 7. This implies the capability to multiplex four channels with a 70% coupling light efficiency within the O-band spectrum, all within a compact footprint area of 8.32 × 22.8 µm.

**Figure 8:**
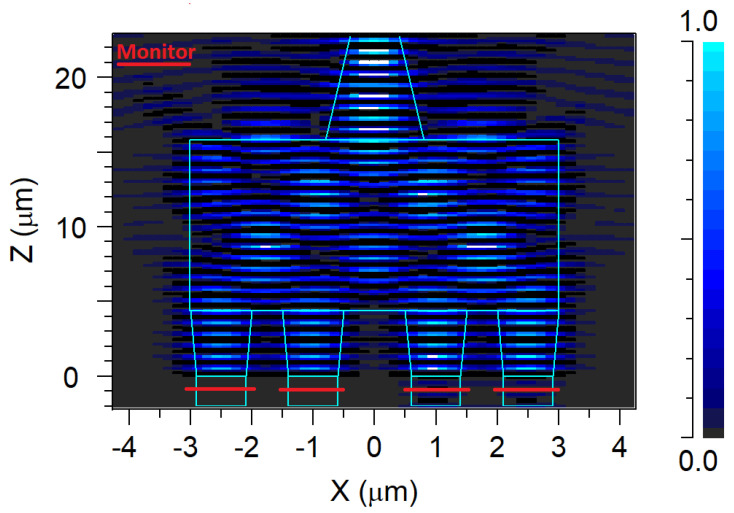
Back reflection FDTD simulation setup.

Reflections of light back into the input waveguide tapers, posing a potential issue for laser sources [41] due to the self-imaging effect, constitute another significant aspect of the MMI coupler. In this investigation, Si_3_N_4_ waveguides were utilized to mitigate the self-imaging-associated back reflection power of the MMI coupler. To evaluate the back reflection power, four monitors were strategically placed within the input waveguide tapers, highlighted in red, as shown in Figure 8, to capture all the returning light from the MMI coupler, as shown in Figure 8. Table 2 presents the back reflection losses determined through FDTD simulation. As anticipated, the Si_3_N_4_ MMI coupler waveguides effectively minimize back reflection across the O-band spectrum without the need for a complex MMI coupler like an angled MMI. The results indicate very low back reflection, making them suitable for use with any standard O-band laser source. In all FDTD simulations, the optimal x, y, and z axis grid sizes were set to 8 nm to ensure satisfactory mesh convergence.

**Figure 9:**
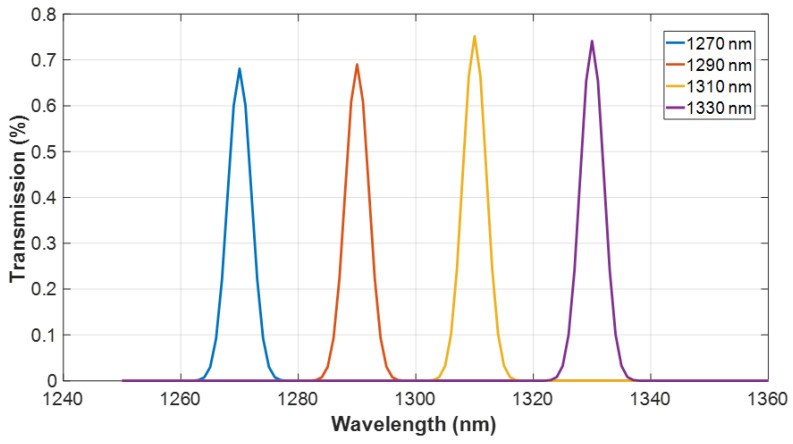
Optical transmission spectrum.

Figure 9 shows the transmission spectrum of our four-channel MMI multiplexer. These spectra are taken for each port separately, and the monitor was placed at the output port. The transmission efficiency is illustrated for each operational wavelength, achieving 68% for 1270 nm, 69% for 1290 nm, 75% for 1310 nm, and 74% for 1330 nm. This figure highlights the multiplexer’s ability to achieve high transmission with minimal insertion loss. Notably, the spectra also reveal excellent channel isolation, attributed to the device’s design that ensures a significant spectral separation between adjacent channels, with 20 nm channel spacing. The sharp, well-defined peaks indicate there is no cross-talk between channels and confirm the device’s high wavelength selectivity, which is crucial for its application in dense wavelength division multiplexing systems.

We present a comprehensive comparison with existing published devices to underscore the innovative aspects and performance superiority of our proposed four-channel MMI multiplexer using a Si_3_N_4_ waveguide. Table 3 compares and focuses on device footprint, number of MMI couplers utilized, range of insertion losses, back reflection levels, and operational spectrum. Notably, our device introduces a remarkably compact footprint of just 6 × 22.6 μm^2^, significantly smaller than any previously reported devices in this category. This is achieved with a single MMI coupler, contrasting sharply with the three or more couplers that are commonly employed in comparable devices. Moreover, our multiplexer maintains competitive insertion losses between 1.27 and 1.67 dB and demonstrates an exemplary back reflection of 40.3 to 41.4 dB, indicating high signal integrity. It operates efficiently across a spectrum range from 1.27 to 1.33 μm, optimized explicitly for the O-band spectrum, making it particularly suitable for data center applications. This table comparison not only highlights our design’s very small footprint and efficiency but also situates our work within the broader context of photonic multiplexers, emphasizing its novelty and potential for significantly impacting densely packed optical data centers.

## 4. Conclusions

This study introduces an innovative four-channel multiplexer designed for the O-band spectrum, utilizing MMI coupler technology with Si_3_N_4_ strip waveguides. The unique aspect of this design lies in the utilization of a single MMI coupler unit for achieving multiplexing of four channels, deviating from the conventional approach of cascaded MMI coupler structures or specially angled MMI structures. The significant advantage of our design, in comparison to other O-band four-channel multiplexers, is its compact footprint size of 8.32 × 22.8 µm. This reduction in size can be harnessed to decrease the total power consumption of photonic transmitter chips operating on WDM technology.

This study unveils the trade-off between power efficiency and compact footprint area, demonstrating the effectiveness of using a single MMI coupler unit for multiplexing four channels. Key parameters for multiplexing the four wavelengths (1270 nm, 1290 nm, 1310 nm, and 1330 nm, spaced 20 nm apart) using Si_3_N_4_ strip waveguides have been identified. The device successfully multiplexes the four signals over a short light propagation of 22.8 µm, achieving a total combining efficiency power of 70% from input sources, with losses ranging from 1.24 to 1.67 dB.

The findings suggest that such a device holds promise for data center applications employing O-band WDM technology, particularly for enhancing data bitrate in transceiver data center chips. In our scenario, our device multiplies the bitrate by four and, when combined with the PAM-4-modulation technique, can further increase it by a factor of eight. Furthermore, the use of Si_3_N_4_ strip waveguides result in a low back reflection loss ranging between 40.3 dB and 41.4 dB, eliminating the need for a complex fabrication process required for angled MMI shapes when using silicon waveguides to minimize back reflection losses.

Importantly, the suggested device design can be easily produced using current fabrication techniques, thanks to the favorable tolerance ranges of ±350 nm and ±300 nm for MMI coupler width and length, respectively. This study provides a valuable foundation for advancing WDM technology with a small footprint, easily integrable into transceiver chips operating in the O-band spectrum to enhance the data bitrate.

## Figures and Tables

**Table 1:** The Si_3_N_4_ refractive index values for the wavelengths operated.

*λ* (nm)	1270	1290	1310	1330
n_Si_3_N_4__	1.9953	1.9951	1.9945	1.9943

**Table 2:** Back reflection results for the operating wavelengths.

Wavelength (nm)	1270	1290	1310	1330
Back Reflection (dB)	−40.7	−41.2	−40.3	−41.4

**Table 3:** Comparison between characteristics of different multiplexer/demultiplexer types.

Multiplexer/Demultiplexer Technology	Footprint (μm^2^)	No. of MMI Couplers	Insertion Loss (dB)	Back Reflection (dB)	Spectrum (μm)	Year of Publication
1 × 4 MMI Si [3]	12 × 1843	3	4.5–9.1	-	1.28–1.58	2018
1 × 4 MMI Si_3_N_4_ [4]	32 × 6630	3	1.98–2.35	40–41	1.53–1.56	2022
1 × 4 MMI SOI [5]	4.83 × 712	4	0.52–1.54	-	0.98–1.55	2018
1 × 8 MMI GaN-Si [6]	12 × 6630	7	0.9–2.12	-	1.53–1.56	2016
1 × 8 MMI SOI [7]	650 × 470	14	2.77–3.77	-	1.27–1.31	2022
1 × 4 MMI Si_3_N_4_ [8]	30 × 1860	6	1.5–2.2	-	1.55–1.565	2015
1 × 4 MMI Si_3_N_4_	8.3 × 22.8	1	1.27–1.67	40.3–41.4	1.27–1.33	Our work

## Data Availability

Data are contained within the article.
